# Coronary heart disease and gut microbiota: A bibliometric and visual analysis from 2002 to 2022

**DOI:** 10.3389/fcvm.2022.949859

**Published:** 2022-09-08

**Authors:** Dan Long, Chenhan Mao, Xinyue Zhang, Yaxuan Liu, Xueli Shangguan, Menglong Zou, Ying Zhu, Xindong Wang

**Affiliations:** ^1^The First Hospital of Hunan University of Chinese Medicine, Changsha, China; ^2^The Third Clinical Medical College, Nanjing University of Chinese Medicine, Nanjing, China; ^3^First College of Clinical Medicine, Shandong University of Traditional Chinese Medicine, Jinan, China; ^4^Affiliated Hospital of Integrated Traditional Chinese and Western Medicine, Nanjing University of Chinese Medicine, Nanjing, China

**Keywords:** gut microbiota, coronary heart disease, bibliometric analysis, visual analysis, trimethylamine-n-oxide

## Abstract

**Background:**

Existing studies have indicated that gut microbiota is closely related to the occurrence and development of coronary heart disease(CHD). Gut microbiota and its metabolites may be important diagnostic markers for CHD in the future and are expected to become new targets for the prevention and treatment of CHD. However, the current studies exploring the link between CHD and gut microbiota are miscellaneous and poorly targeted, without bibliometric analysis available.

**Objective:**

The purpose of this research was to perform a bibliometric and visual analysis of published papers on the relationship between CHD and gut microbiota. The study also sought to identify principal authors, institutions, and countries to analyze the research status and trends of gut microbiota research in the field of CHD.

**Methods:**

The Web of Science Core Collection (WoSCC) database was searched for publications on CHD and gut microbiota between 2002 and 2022. CiteSpace 5.8. R1, VOSviewer 1.6.16, and Microsoft Excel 2019 software tools were utilized to perform this bibliometric analysis and visualization.

**Results:**

There were 457 qualified publications found in total, with the annual number of publications increasing. The United States dominated in this field. Hazen, Stanley l was the author of the most papers. Cleveland Clinic published the most papers of any institution. The six main clusters’ specific characteristics were discovered through analysis of the co-occurrence of keywords: inflammation, diet, trimethylamine n-oxide, metabolism, cardiovascular disease, and myocardial infarction. Newly emerging research has focused predominantly on gut microbiota metabolites and recent strategies for intervention in coronary atherosclerosis.

**Conclusion:**

These results provided a useful perspective on current research and future prospects for the research on the link between CHD and gut microbiota, which may help researchers to select suitable collaborators and facilitate their research to elucidate the underlying molecular mechanisms of CHD, including the causes, prevention, and treatment.

## Introduction

Coronary heart disease (CHD), with its high morbidity and mortality, poses a heavy burden on the global healthcare system (1). Although great progress has been made in the coronary artery blood revascularization technology, the promotion of cardiac rehabilitation programs, and the control of risk factors for CHD, the mortality of CHD remains high and CHD is still a clinical problem that cannot be ignored (2–4). There is a pressing need to discover new diagnostic markers of CHD and develop new treatments to solve the current dilemma.

Gut microbiota is of great significance to human health, with its functions of digesting food, synthesizing essential vitamins, stimulating and regulating the immune system, supporting intestinal function, etc. (5). Gut microbiota in a healthy organism is relatively stable, and the symbiotic relationship with the host is beneficial to the growth of symbiotic bacteria and can also inhibit the overgrowth of conditioned pathogenic bacteria (6). Numerous clinical studies have found that the increased risk of CHD may be related to changes in the structure of the gut microbiota (7). Dysbacteriosis may be an important risk factor for the development of CHD, and gut microbiota may become an important diagnostic marker for CHD in the future (8).

Citespace and VOSviewer are bibliometric and visualization software that can play an important role in analyzing the current state of scientific research, detecting the frontiers of the discipline and selecting research directions (9, 10). Bibliometrics can provide us with the most influential countries, institutions, authors of the field we wanted to look into through related publications, supplying potentially valuable information to researchers qualitatively and quantitatively (11). However, there have been few bibliometric studies on CHD and gut microbiota. Based on bibliometric analysis, we formed a knowledge graph of CHD and gut microbiota to provide implications for researchers to discover new topics and directions.

## Materials and methods

### Data selection

**FIGURE 1:**
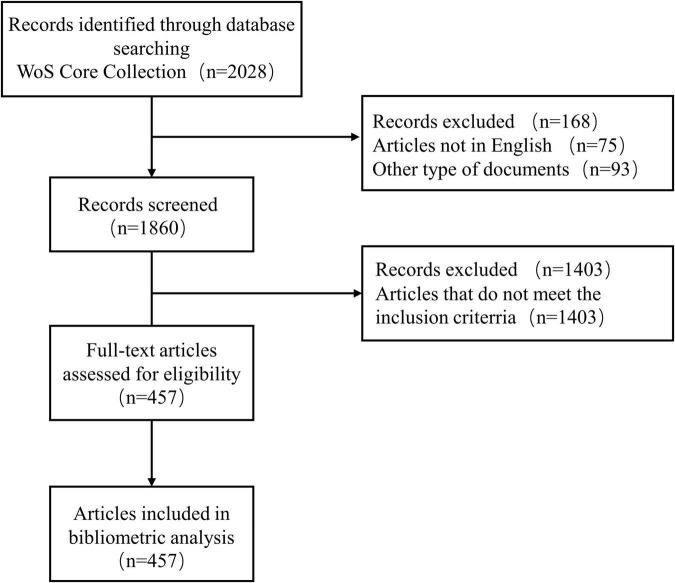
Flowchart of literature identification and selection.

We screened the Web of Science Core Collection (WoSCC, Clarivate) for the period from 1st January, 2002 to 31st July, 2022 to identify publications related to CHD and gut microbiota. Details of the search terms are provided in Supplementary Table 1. Only publications published in English are taken into account. Two independent reviewers (Dan Long, Chenhan Mao) reviewed and screened the title and abstract; a third reviewer (Xinyue Zhang) resolved any disagreements. The flowchart of literature identification and selection was displayed in Figure 1.

### Data analysis and visualization

From articles that matched the criteria, key information such as title, author, country/territory, institution, keywords, and publication year was extracted. The above variables were processed and visualized using Microsoft Excel 2019, CiteSpace 5.8.R1 software, and VOSviewer (Version 1.6.16, Leiden University, Netherlands).

The “full record and citations” of these records are extracted into the CiteSpace 5.8.R1 software in the format of “plain text.” No duplicate records were found by using the software’s native duplicate check function. Keywords with the same meaning were merged in the exported data. For example, the keywords “intestinal microbiota” and “gut microbiota” will be merged into “gut microbiota.” Keywords were specified uniformly, such as specifying “trimethylamine-n-oxide” as “trimethylamine n-oxide.” From keywords burst detection and other aspects, CiteSpace 5.8 R1 software was utilized to conduct bibliometric and visual knowledge graph analysis. The parameters of CiteSpace were as follows: time span (2002–2022), years per slice (2), selection criteria (Top 50), Pruning: Pathfinder, Pruning sliced networks, Pruning the merged network. VOSviewer was applied to perform the network analysis of the frequent keywords. The parameters of VOSviewer were as follows: the minimum number of occurrences of a keyword was 7.

## Results

### General information

**FIGURE 2:**
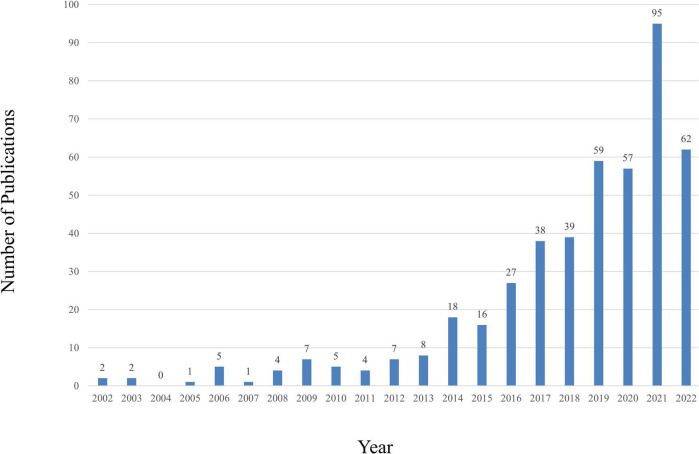
The number of annual publications relating to research about CHD and gut microbiota from 2002 to 2022.

A total of 2,028 publications from WoSCC were identified in the literature search. A total of 457 studies, including 295 articles and 162 reviews, were selected for further data extraction based on the titles and abstracts of papers. Figure 1 shows a flowchart of the literature selection process. We created a histogram (Figure 2) to represent the number of annual publications over the last 20 years, which indicated the development trend of research in this field. The number of publications has been increasing in recent years, indicating growing attention and interest of researchers in this field. From 2002 to 2013, the number of publications on CHD and gut microbiota does not exceed 8. But since 2014, this number has begun to increase, reaching a peak of publication in the past 3 years. Overall, these data suggest that research on CHD and gut microbiota has gained widespread attention. In particular, significant progress has been made in this field since 2019.

### Distribution of authors

**FIGURE 3:**
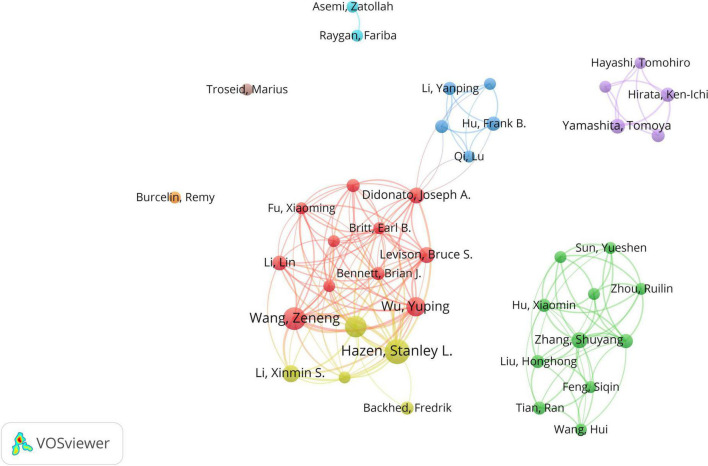
The network of authors contributed to research about CHD and gut microbiota. In the network, author contribution is reflected by node size. The connection strength is reflected by the thickness of line.

The top 10 authors respectively had over 6 papers on CHD and gut microbiota, 5 of whom published their first related peper in 2013 (Table 1). Stanley L Hazen had the most publications (25 publications) and cooperated more with Didtonato, Joseph A, Lin Li, Yuping Wu, and W. H. Wilson Tang. The author collaboration network visualization was performed by the VOSviewer, as presented in Figure 3. The collaboration between authors was close, while the centrality of each author was much less than 0.1. A co-cited relationship exists when two or more authors’ papers were cited by another paper or multiple papers simultaneously. Ten of the 335 authors were cited more than or equal to 70 times in this study and the centrality of Wang ZN, Karlsson FH, Zhu WF, Koeth RA and Tang WHW was over 0.4 (Table 2).

**TABLE 1:** Top 10 authors on CHD and gut microbiota.

Rank	Author	Count	Year	Centrality	Country
1	Stanley L. Hazen	25	2013	0.02	United States
2	Zeneng Wang	20	2013	0.01	United States
3	Tang WHW	17	2013	0.02	United States
4	Yuping Wu	12	2013	0	United States
5	Xinmin S. Li	10	2015	0	United States
6	Shuyang Zhang	10	2019	0	China
7	Hanyu Li	7	2019	0	China
8	Xiaomin Hu	6	2019	0	China
9	Lin Li	6	2013	0.02	United States
10	Frank B. Hu	6	2016	0.02	United States

**TABLE 2:** Top 10 co-cited authors on CHD and gut microbiota.

Rank	Co-cited author	Citation	Centrality
1	Tang WHW	233	0.41
2	Wang ZN	217	0.87
3	Koeth RA	173	0.43
4	Zhu WF	98	0.47
5	Karlsson FH	93	0.59
6	Jie ZY	87	0.19
7	Li J	77	0.02
8	Turnbaugh PJ	74	0.31
9	Qin JJ	70	0.36
10	Cani PD	70	0.24

### Distribution of countries/territories and institutions

**FIGURE 4:**
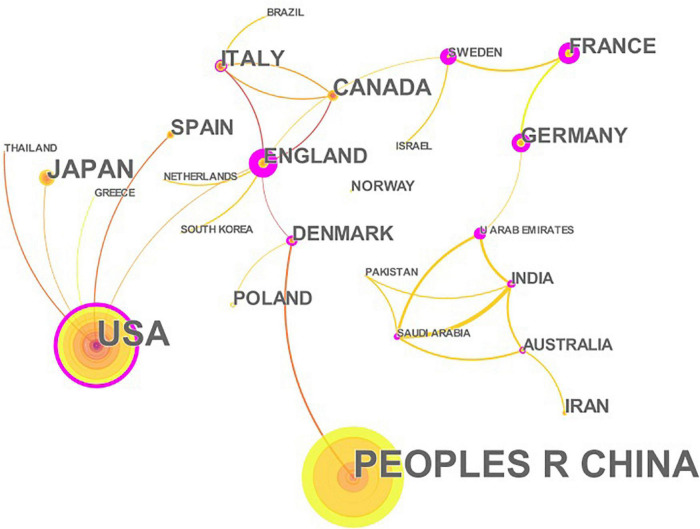
The network of countries/territories engaged in the research about CHD and gut microbiota. In the networks, the larger the node was, the more contribution the country/territory had made to that field. The nodes with higher centrality (>0.1) are highlighted with purple rings. The color of the line represents the time of first co-occurrence. The thicker the line is, the greater the connection strength is (calculation method based on cosine).

**FIGURE 5:**
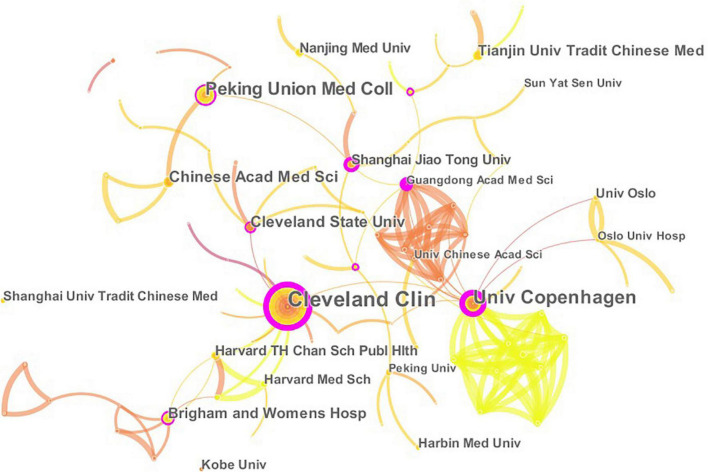
The network of institutions engaged in the research about CHD and gut microbiota. In the networks, the larger the node was, the more contribution the institution had made to that field. The color of the line represents the time of first co-occurrence. The thicker the line is, the greater the connection strength is (calculation method based on cosine).

A total of 457 papers on CHD and gut microbiota have been co-authored by 835 institutions from 61 countries/territories. As depicted in Figure 4 (node = 25, line = 28), China had the most publications (153), followed by the United States (119), Japan(28), Canada (26), Italy (23), England (22), Germany (21), Denmark (18), France(18), Spain (18), and Iran(15). Research in this field has exploded in the United States since 2002, while it has only emerged in China since 2012. The importance of nodes in a network is measured using centrality. In the visualization network, nodes with a centrality of more than 0.1 were called key nodes and usually highlighted with a purple ring. The links between Countries or Institutions were reflected by the lines between the nodes. The top countries for Centrality were England (1.39), Sweden (0.97), France(0.81), Germany (0.74), and United Arab Emirates (0.65). The institution collaboration network was demonstrated in Figure 5 (Nodes = 117, Links = 168). The top five institutions with the most publications were Cleveland Clinic (28), University of Copenhagen (15), Peking Union Medical College (14), Chinese Academy of Medical Sciences (10), and Cleveland State University (10). The centrality of Cleveland Clin and Univ Copenhagen was far more than 0.1, and there was close cooperation between different institutions.

### Analysis of co-cited and cited references

Co-cited references are two or more references that were cited by another or more papers simultaneously. To analyze papers with high co-citation frequency can contribute to understanding the foundations of disciplinary research. The 457 publications on CHD and gut microbiota in the last 20 years included 143 co-cited references, 5 of which were cited over 80 times in total (Table 3). We also listed Top 5 cited references on CHD and gut microbiota in Table 4.

**TABLE 3:** Top 5 co-cited references on CHD and gut microbiota.

Rank	Author	Year	Title	Citation	Source	Reference
1	Tang et al.	2013	Intestinal microbial metabolism of phosphatidylcholine and cardiovascular risk	149	NEW ENGLAND JOURNAL OF MEDICINE	(12)
2	Koeth et al.	2013	Intestinal microbiota metabolism of L-carnitine, a nutrient in red meat, promotes atherosclerosis	144	NATURE MEDICINE	(13)
3	Wang et al.	2011	Gut flora metabolism of phosphatidylcholine promotes cardiovascular disease	100	NATURE	(14)
4	Zhu et al.	2016	Gut Microbial Metabolite TMAO Enhances Platelet Hyperreactivity and Thrombosis Risk	95	CELL	(15)
5	Jie et al.	2017	The gut microbiome in atherosclerotic cardiovascular disease	87	NATURE COMMUNICATIONS	(16)

**TABLE 4:** Top 5 cited references on CHD and gut microbiota.

Rank	Author	Year	Title	Citation	Source	Reference
1	Wang et al.	2011	Gut flora metabolism of phosphatidylcholine promotes cardiovascular disease	3657	NATURE	(14)
2	Koeth et al.	2013	Intestinal microbiota metabolism of L-carnitine, a nutrient in red meat, promotes atherosclerosis	2534	NATURE MEDICINE	(13)
3	Tang et al.	2013	Intestinal microbial metabolism of phosphatidylcholine and cardiovascular risk	1900	NEW ENGLAND JOURNAL OF MEDICINE	(12)
4	Karlsson et al.	2012	Symptomatic atherosclerosis is associated with an altered gut metagenome	700	NATURE COMMUNICATIONS	(17)
5	Koren et al.	2011	Human oral, gut, and plaque microbiota in patients with atherosclerosis	820	PROCEEDINGS OF THE NATIONAL ACADEMY OF SCIENCES OF THE UNITED STATES OF AMERICA	(18)

### Analysis of keywords

**FIGURE 6:**
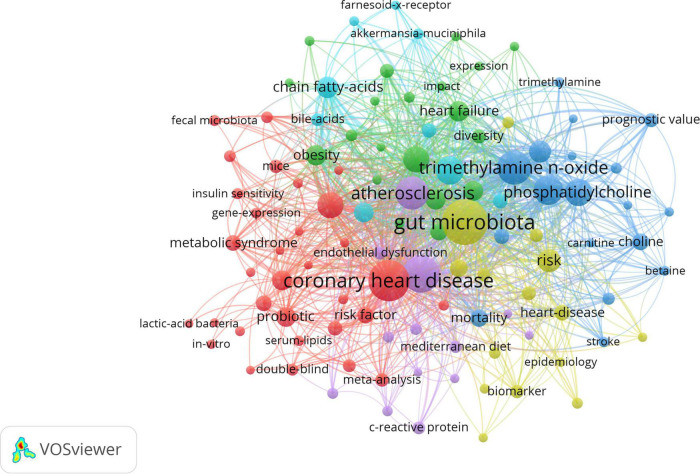
The network visualization of keywords. The size of each circle represents the weight of a keyword. The distance between two circles indicates the relatedness between the two circles. The stronger the relatedness, the shorter the distance. The color of the circles represents the respective cluster class.

The analysis of keywords provided us with a glimpse not only into the subject matter of the article but also into the hotspots of a particular area of research. The top 20 keywords with the highest frequency were presented in Table 5. The VOSviewer was employed to visualize the keywords’ network, as demonstrated in Figure 6. The clusters of 6 colors represented 6 research directions. The keywords of the red cluster were coronary heart disease, inflammation, cholesterol, risk factor, probiotic, metabolic syndrome, meta-analysis, hypercholesterolemia, etc. The keywords of the green cluster were diet, heart failure, hypertension, infarction, and regulatory t-cells. The keywords of the blue cluster were trimethylamine n-oxide, metabolism, phosphatidylcholine, stroke, red meat, etc. The keywords of the yellow cluster were gut microbiota, cardiovascular risk-factors, acute myocardial infarction, biomarker, nutrition, etc. The keywords of the purple cluster were atherosclerosis, endothelial dysfunction, c-reactive protein, mediterranean diet, low density lipoprotein, etc. The keywords of the dark blue cluster were myocardial infarction, bile acids, blood pressure, chain fatty acids, etc.

**TABLE 5:** Top 20 keywords on CHD and gut microbiota.

Rank	Keywords	Count	Centrality	Rank	Keywords	Count	Centrality
1	Gut microbiota	233	0.08	11	Inflammation	70	0.04
2	Coronary heart disease	199	0.03	12	L-carnitine	46	0.03
3	Cardiovascular disease	154	0.08	13	Chain fatty acid	42	0.12
4	Trimethylamine n-oxide	129	0.08	14	Heart failure	42	0.18
5	Atherosclerosis	123	0.05	15	Obesity	42	0.12
6	Metabolism	96	0.01	16	Probiotic	41	0.2
7	Risk factor	93	0.07	17	Blood pressure	39	0
8	Phosphatidylcholine	80	0.15	18	Diet	37	0.08
9	Myocardial infarction	79	0.11	19	Cholesterol	31	0.1
10	Microbiota	73	0	20	Mortality	31	0.03

**FIGURE 7:**
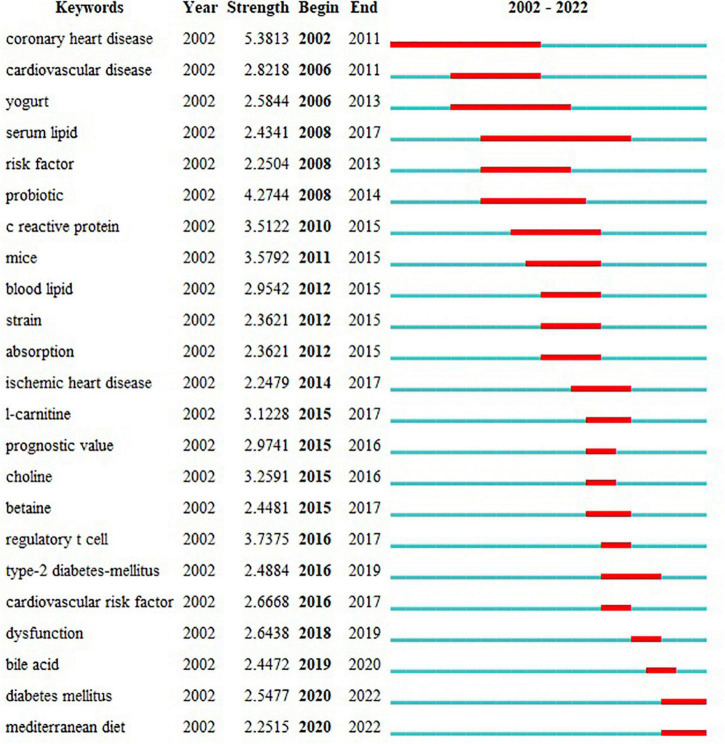
Top 23 keywords with the strongest citation bursts. Begin and End represent the beginning and end years of keyword emergence respectively. Strength indicates the intensity of the cited change. Each red or blue bar represents the time interval, and a single bar is equal to one year. The red bar especially represents citation burst.

The keyword citation burst, performed by CiteSpace, can show how research hotspots have shifted over time so as to determine potential trends and cutting-edge research. The minimum duration was set to 2 years, γ = 0.7. 23 keywords with the strongest citation bursts were identified and grouped based on the year the citation outbreak started (Figure 7). The citation intensity value indicates the intensity of the citation burst.

## Discussion

### General information

In terms of posting trends, we found that publications on research in this area had increased rapidly since 2002. Especially after 2019, there has been a gradual increase in the number of publications related to this field, thus indicating that this field will continue to be of great interest to researchers in the next years.

The author’s collaborative network analysis was useful in identifying influential research groups and potential partners, building collaborative relationships for researchers. As demonstrated in Figure 3, many authors contributed to this field, while their collaboration needs to be increased. The top 10 authors on CHD and gut microbiota were listed in Table 1. We found the centrality of authors was far less than 0.1, showing that their cooperation is not close enough in the field. China published the most papers, but had a centrality of less than 0.1. Besides, American authors have been studying CHD and gut microbiota since 2002. The United States had more prolific writers, with 7 of the top 10 authors working in the United States. Therefore, the United States may be a leader in CHD and gut microbiota research. As depicted in Figure 5, most of the well-published institutions are based in China. To promote research and development of this field, it is strongly recommended that researchers and research institutions from various countries actively collaborate and exchange ideas.

As shown in Table 3, the top 5 co-cited references mainly covered the following intriguing aspects. First, elevated levels of trimethylamine-N-oxide (TMAO), a metabolite of gut microbiota, are associated with an increased risk of major adverse cardiovascular events (12–14) due to platelet hyperreactivity and enhancing thrombosis (15). The second aspect was a genomic correlation of gut microbiota in patients with CHD (16). However, it remains a challenge to treat patients with CHD from the perspective of gut microbiota.

### Current research hotspots

We categorized studies on CHD and gut microbiota into three key hotspots based on keyword co-occurrence analysis, as described below.

**(I) Metabolites of gut microbiota influence the progression of CHD**

The key point in the mechanism of gut microbiota associated with the pathogenesis of CHD is that dysbiosis and its metabolites can lead to coronary atherosclerosis. Metabolites of gut microbiota (Table 6), such as TMAO, SBAs, and SCFAs, are associated with the development of CHD. TMAO is undoubtedly a typical metabolite of gut microbiota, and numerous studies have shown its relevance to a wide range of cardiovascular diseases (CVDs) (33). For example, a prospective study showed that a long-term increase in TMAO was associated with a higher risk of CHD and that repeated assessment of TMAO over a 10-year period facilitated the identification of people at high risk of CHD (34). In addition, the SYNTAX score of patients with stable CHD was independently correlated with TMAO (35). All of the above studies suggest that TMAO could serve as a novel serum marker, or as a potential target for intervention. There has also been a proliferation of research into the mechanisms by which TMAO is associated with CHD. For example, the highly cited paper by Weifei Zhu (15) demonstrated that TMAO enhanced the stimulus-dependent Ca^2+^ signaling pathway and thus played a role in regulating platelet hyperresponsiveness and thrombogenic potential through *in vitro* platelet agglutination experiments and animal studies. TMAO also has a critical impact on in the process of atherosclerosis by means of immunity (36), inflammation (37), cholesterol metabolism (38), etc. In conclusion, research on TMAO and CHD has never stopped. It has been, is, and will continue to be a hot topic. Lipopolysaccharide (LPS), also known as endotoxin, is a complex of lipids and polysaccharides. LPS derived from gut microbes can bind to transmembrane receptors and activate immune cells to release inflammatory mediators, thus increasing microvascular permeability, and causing platelet adhesion to blood vessel walls. Another study showed that LPS initiates the transcription of inflammatory factors through the TLR4-MyD88-NFκB pathway, to induce the release of inflammatory factors and trigger chronic low-grade inflammation, which plays an important role in the progression of CHD (39). The team of Stanley L. Hazen of the Cleveland Clinic found that phenylalanine was associated with an increased risk of CVD in T2DM patients through metabolomic analysis. This substance is metabolized by gut microbiota to form phenylacetylglutamine (PAGln). The mechanism of PAGln driving cardiovascular risk is mainly related to fostering platelet responsiveness and thrombosis potential via adrenergic receptors (32).

**TABLE 6:** Main metabolites of gut microbiota related to CHD.

Metabolites of gut microbiota	Possible mechanism of action	References
TMAO	Upregulating scavenger receptor expression in macrophages Increasing the expression of cellular Heat shock protein (HSPs) Increasing the expression of proinflammatory cytokine Increasing plasma cholesterol levels Contributing to platelet hyperreactivity and enhancing the potential for thrombosis	(14, 19, 20)
Short-chain fatty acids (SCFAs)	Regulating renin secretion and blood pressure Regulating the expression of intercellular adhesion molecules and improving vascular endothelial function Lowering energy intake by promoting the production of anorectic hormones Stimulating the synthesis of bile acids Reducing the expression of pro-inflammatory factors to regulate the occurrence of inflammation	(21–26)
Secondary bile acids (SBAs)	Regulating systemic lipid and glucose metabolism Reducing the release of pro-inflammatory factors	(27, 28)
Lipopolysaccharide (LPS)	Inducing formation of foam cell and lipid accumulation to accelerate AS Exerting a pro-inflammatory effect leading to increased blood pressure Increasing platelet activation	(29–31)
Phenylacetylglutamine (PAGln)	Fostering platelet responsiveness and thrombosis potential via adrenergic receptors	(32)

**(II) The effect of gut microbiota on CHD is two-fold**

Increased rates of harmful flora can promote the development of CHD, but probiotics can effectively control the risk factors for CHD and thus slow down the progression of CHD. This means that the effect of gut microbiota on CHD is two-fold. A clinical study found that daily doses of Lactobacillus plantarum 299v for 6 weeks in patients with CHD significantly improved cardiovascular endothelial function and reduced systemic inflammation (40). The relationship between Short-chain fatty acids (SCFAs), secondary bile acids (SBAs), and CHD is one of the main directions currently being explored by the academic community. SCFA is produced by gut microbiota through the fermentation and breakdown of carbohydrates and other diets, and has been confirmed to modulate the body’s immune response to improve the prognosis of myocardial infarction and repair the heart (41). Its association with cardiovascular risk factors such as hypertension, kidney disease, obesity and diabetes (42, 43) has been established in animal models, involving mechanisms such as regulating the expression of intercellular adhesion molecules and improving vascular endothelial function, reducing the expression of pro-inflammatory factors to regulate the occurrence of inflammation and so on. However, a clear link between SCFA and cardiovascular disease risk or mortality in humans cannot be confirmed at this time due to the lack of strong support from independent replicated clinical studies (44). Bile acids are generated by the gut microbiota to generate SBAs. Free SBAs have pleiotropic and hormonal activities and are effective anti-inflammatory molecules. Studies have shown that the bile acid receptor TGR5 suppresses the inflammatory response of macrophages by interfering activity of NF-κB (28). Another clinical study found that lithocholic acid, a type of secondary bile acid, may be an independent predictor of coronary atherosclerosis (45). To sum up, In-depth research on the beneficial flora helps to offer insight on microbiota-targeted therapy options in CHD management.

**(III) Dietary modification is an important way to decelerate the progression of CHD**

Dietary structure exerts a vital effect on the development of CHD, offering new low-cost, easy-to-manage options for the management and treatment of CHD. Gut microbiota may reveal a potential underlying mechanism of dietary therapy for CHD. The Mediterranean diet is a style of eating that is based on an abundance of vegetables, fresh fruit, fish, grains and cereals, olive oil, and other oxidant-rich foods (46). It can achieve cardioprotective effects through mechanisms such as lowering blood pressure and insulin resistance, improving blood lipids, and anti-inflammation (47). Studies have shown that gut microbiota mediates the beneficial effects of the Mediterranean diet on cardiometabolic disease risk factors (48, 49). In addition, reducing the intake of TMA nutrients such as red meat indirectly adjusts gut microbiota and reduces TMAO, which is advantageous for the prevention and treatment of CHD. A prospective nested case-control study showed that women with a higher intake of red meat had higher L-carnitine levels and a higher incidence of CHD (50). There is a proven association between red meat consumption and high mortality risk for CHD, which will not be affected by lifestyle and genetic risk factors (51). Other studies have indicated that vegetarians have lower fasting TMAO levels than omnivores and have a significantly lower ability to synthesize TMAO after oral carnitine (13). Similarly, a low-calorie diet may also reduce the level of TMAO by reducing the intake of TMAO precursors (52). It is well known that a high-salt diet leads to increased cardiovascular risk. Interestingly, high-salt diets have also been reported to cause imbalances in gut microbiota and metabolism of bile acid, thus leading to elevation of lipids (53). Another animal experiment found a high-salt diet causes elevated TMAO in the circulation and brain, leading to sympathetic arousal and increased blood pressure (54). These results certainly reveal the significance of a low salt diet in the prevention and treatment of CHD from the perspective of gut microbiota. Although all the special diets described above can regulate gut microbiota to help treat CHD, some factors such as poor patient compliance and personal preference may make it difficult to formulate nutritional prescriptions.

### Future frontiers

Based on our keyword emergent analysis of research on CHD and gut microbiota, we predict 2 research themes that will play a significant role in the future, as shown below.

(I) Novel intervention strategies to modulate gut microbiota and its metabolites in patients with CHD

With advances in research into the relationship between CHD and gut microbiota, we put forward several possible hotspot treatment strategies for the future, as described below. Natural compounds (Table 7) have long been effective in the treatment and prevention of CHD and its risk factors, and there is a growing body of research into natural products and their mechanisms of action (55, 56). Resveratrol, a natural phytoantitoxin with probiotic effects, can reduce AS to prevent CHD by reshaping the gut microbiota to regulate TMAO synthesis and bile acids (BAs) metabolism (57). The experiments have confirmed that the mechanism of naringin’s prevention of atherosclerosis is related to gut microbiota remodeling (58). Chinese medicine also belongs to natural products, and numerous studies have revealed that the multi-component and multi-target characteristics of Chinese medicine are unique in intervening in CHD through the regulation of gut microbiota. Ligustrum robustum may attenuate the development of AS by reducing serum TMAO levels and increasing fecal BA excretion through gut microbial regulation (59). The aqueous extract of Mulberry Leaf can promote the fermentation of gut microbiota and excretion of BA by the production of SCFA to delay the progression of CHD (60). What’s more, research into fecal transplantation has attracted our attention. Fecal transplantation is an effective means of introducing functional flora from donor feces into the recipient to help rebuild the structure of gut microbiota and maintain microbial homeostasis, which may be more effective than probiotics in restoring gut microbiota in the long term. This method has been approved by the FDA for the treatment of recurrent Clostridioides difficile infection (61). One investigator transplanted fecal bacteria with a high TMAO level into normal mice and found an increased risk of atherosclerosis in these mice, which led to the hypothesis that gut microbiota may serve as a novel therapeutic target for CHD (62). However, there are many risks and controversies associated with fecal transplantation. The active components in the donor fecal bacteria can’t be clearly identified with the current technology and transplantation may cause, for example, the introduction of endotoxins and rejection reactions. New intestinal complications and even more serious adverse reactions can occur in immunocompromised patients, so further research into this strategy is needed. Further research into this strategy is therefore needed.

**TABLE 7:** Natural compounds intervening CHD through the regulation of gut microbiota.

Natural compounds	Structural changes in gut microbiota	Possible mechanism of action	References
Resveratrol	Ruminococcaceae_uncultured↓ Prevotella↓ Bacteroides↑ Akkermansia↑ Lactobacillus↑	Reshaping the gut microbiota to regulate TMAO synthesis and bile acids (BAs) metabolism	(57)
Seaweed	Lactobacilli↑ Bacteroides↓	Promoting the production of SCFAs	(63)
Berberine	Akkermansia↑ Bacteroides↑ Lachnospiraceae_NK4A136_group↑ Eubacterium↑	Improving hypercholesterolemia and systemic inflammation; Inhibiting the production of TMA/TMAO and choline-to-TMA conversion	(64, 65)
Ligustrum robustum	Actinobacteria↑ Bifidobacterium↑ Rikenellaceae_R9_gut_group↑ Prevotellaceae_UCG-001↓ Lachnospiraceae_NK4A136_group↓ uncultured_Bacteroidales_bacterium↓ Lachnospiraceae_FCS020_group↓ Odoribacter↓ Oscillibacter↓	Reducing serum TMAO levels and increasing fecal BA excretion	(59)
Ginseng	Firmicutes↓ Bacteroidetes↑	Repairing the intestinal barrier and alleviating metabolic endotoxemia related inflammation	(66)
Mulberry Leaf	Leptotrichia↑ Bacteroidetes↑	Promoting the fermentation of gut microbiota and excretion of BA by the production of SCFA	(60)
Saussurea involucrata	Not mentioned	Stimulating intestinal bacteria to produce short chain fatty acids *in vitro*, further contributing to the effect in myocardial ischemia	(67)
naringin	Bacteroides↓ Bifidobacterium↓ Clostridium↓ Eubacterium↑	Modulating the abundances of bile salt hydrolase- and 7α-dehydroxylase-producing bacteria, promoting bile acid synthesis from cholesterol	(58)
Lingonberry	Bacteroides↑ Parabacteroides↑ Clostridium↑	Lowering plasma total cholesterol and LDL-VLDL, but increasing cecal proportion of propionic acid	(26)

(II) The specific mechanism of action of gut microbiota affecting CHD remains to be elucidated

Many scholars have studied the changes of gut microbiota in patients with CHD, as shown in the Table 8.

**TABLE 8:** CHD-related structural changes in the gut microbiota.

Year	Researchers	CHD-related structural changes in gut microbiota	Methods	Conclusion	Reference
2016	Emoto et al.	Lactobacillales↑ Phylum Bacteroidetes↓	Terminal restriction fragment length polymorphism (T-RFLP) and 16S rDNA	The incidence of CAD was linked with an alteration of gut microbiota	(68)
2017	Emoto et al.	Lactobacilli↑ Bifidobacterium↓ Prevotella↓	T-RFLP and 16S rRNA	T-RFLP is a well-established method and revealed the characteristic patterns of gut microbiota to distinguish CAD patients from healthy controls. Gut microbiota may have a potential to be a diagnostic marker of CAD	(69)
2017	Cui et al.	Phylum Firmicutes↑ Phylum Bacteroidetes↓	High-throughput sequencing and 16S rRNA	The diversity and composition of gut flora were different between CHD patients and healthy controls. The incidence of CHD may be associated with an alteration of gut microbiota	(70)
2017	Jie et al.	Streptococcus↑ Escherichia↑ Bacteroides↓ Prevotella↓	Metagenomics	It is promising to predict diseases such as CHD by gut microbes. Diabetes, obesity, and so on, as cardiovascular risk factors, share many concordances on the gut microbiota	(16)
2018	Zhu et al.	Proteobacteria↑ Actinobacteria↑ Firmicutes↓ Bacteroidetes↓	16S rRNA	Functions such as amino acid metabolism, propanoate metabolism were found to be enhanced in CAD patients. The amount of richness and diversity of CAD microbiomes decreased	(29)

However, there are still some uncertainties to be explored in the study of the mechanisms of CHD and gut microbiota. First, the correlation between concentrations of TMAO and cardiovascular disease is controversial, and whether it is due to other diseases remains to be investigated. It has been reported that plasma TMAO concentrations in people at high risk of CHD are susceptible to renal function and poor metabolism and that the levels are not associated with a history of CHD, symptoms, or the incidence of cardiovascular events (71). Perhaps only in cases of HF, CKD, or CHD comorbid disease, there will be elevated concentrations of TMAO which can promote inflammation, atherosclerosis, cardiac dysfunction, and remodeling (72). Second, we found that fish diets are rich in TMA, but fish consumption and fish oil supplementation may have a positive impact on cardiovascular health (73). The fish diet is rich in long-chain omega-3 polyunsaturated fatty acids (PUFAs), which have anti-inflammatory, antioxidant, and anti-thrombotic effects. However, most published randomized controlled trials have not shown a benefit of omega-3 PUFAs as primary prevention of coronary heart disease in patients with cardiovascular risk factors, and the mechanisms remain to be investigated (74). Existing studies on gut microbiota and CHD are not uniform and standardized because of platform, sample and other limitations. In response to the above discussion, we suggest that the research on CHD and gut microbiota is on the rise and needs to be improved in terms of breadth and depth. Firstly, more standardized, scientific basic, animal, and clinical experiments are needed for further in-depth research. Secondly, in addition to CHD, other diseases also deserve to be studied and alterations in the species of specific gut microbiota and the mechanisms of diseases should be involved to determine specific causal relationships. Last but not least, due to the extreme complexity of the gut microbiota and its metabolites, a multidisciplinary approach and emerging research tools should be used in future studies to further explore the mysteries. The development of new genome sequencing technologies is expected to to better understand the gut microbiota.

This study has some limitations that should be noted. Firstly, related publications from high-quality databases were not included due to software limitations, which might have resulted in an imperfect literature screening. Secondly, data for the full year 2022 could not be included in this study to allow for a comprehensive analysis in terms of annual posting statistics. Finally, there is a certain amount of subjectivity in the manual selection process of the literature.

## Conclusion

Our study identified 457 publications on CHD and gut microbiota between 2002 and 2022, revealing influential countries, institutions, and authors that made significant contributions to this field. Moreover, we concentrated on specific topics in order to investigate research trends. TMAO remained the current research object in this field. Dietary therapy offered a viable avenue for further research into prevention and treatment for CHD, while natural products and fecal transplantation may be the focus of future research.

## Data availability statement

The original contributions presented in this study are included in the article/Supplementary material, further inquiries can be directed to the corresponding authors.

## Ethics statement

Ethical review and approval was not required for this study in accordance with the local legislation and institutional requirements.

## Author contributions

DL, CM, and XZ contributed to concept, design, and manuscript writing, were responsible for literature search, data acquisition, data analysis, and statistical analysis. DL, CM, XZ, and YL contributed to data supervision and edited the manuscript. XS and MZ provided valuable suggestions for data analysis and figure design. YZ and XW reviewed the manuscript. All authors read and approved the final manuscript.
