# Supplementary material for: Coronary heart disease and gut microbiota: A bibliometric and visual analysis from 2002 to 2022
Source: Front Cardiovasc Med. 2022 Sep 8;9:949859. doi: 10.3389/fcvm.2022.949859 (PMC9493042; doi:10.3389/fcvm.2022.949859)
Supplement: Supplementary file 1 [file Table_1.docx]

**TABLE 1 Details of search strategy**

| Number | Search term |
| --- | --- |
| 1 | TS=(coronary heart disease) |
| 2 | TS=(coronary artery disease) |
| 3 | TS=(coronary disease) |
| 4 | TS=(silent myocardial ischemia) |
| 5 | TS=(angina) |
| 6 | TS=(stenocardia) |
| 7 | TS=(myocardial infarction) |
| 8 | TS=(ischemic cardiomyopathy) |
| 9 | TS=(ischemic heart disease) |
| 10 | TS=(coronary death) |
| 11 | TS=(gut) |
| 12 | TS=(intestin*) |
| 13 | TS=(gastrointestin*) |
| 14 | TS=(gastro-intestin*) |
| 15 | TS=(microbiot*) |
| 16 | TS=(microbiome*) |
| 17 | TS=(ﬂora) |
| 18 | TS=(microﬂora) |
| 19 | TS=(bacteria) |
| 20 | TS=(prebiotic) |
| 21 | TS=(probiotic) |
| 22 | TS=(antibiotic) |
| 23 | TS=(dysbiosis) |
| 24 | 1 OR 2 OR 3 OR 4 OR 5 OR 6 OR 7 OR 8 OR 9 OR 10 |
| 25 | 11 OR 12 OR 13 OR 14 |
| 26 | 15 OR 16 OR 17 OR 18 OR 19 |
| 27 | 25 AND 26 |
| 28 | 20 OR 21 OR 22 OR 23 OR 27 |
| 29 | 24 AND 28 |
| TS = title, abstract, author keywords and keywords plus. | |
| * = any ending to the word | |
